# Enhanced Postoperative Pain Management: A Comparative Analysis of Ultrasound-Guided Quadratus Lumborum Block Versus Intraperitoneal and Periportal Bupivacaine Infiltration Following Laparoscopic Cholecystectomy: A Randomized Double-Blind Study

**DOI:** 10.5812/aapm-159545

**Published:** 2025-03-16

**Authors:** Ashraf Nabil Saleh, Sherif F. Ibrahim, Yasmeen A. Sayed, Mohammed Mawad Alsaid A, Karim Ahmed Sedky Abdelrahman, Mohamed Mahmoud Abdelaziz Aly, Mohammed Abdulgadir Ageel, Ahmed Gamal Salah Elsawy, Mohamed Elsayed Mahmoud, Amr M. Hilal

**Affiliations:** 1 Department of Anesthesiology, Intensive Care and Pain Management, Faculty of Medicine, Ain Shams University, Cairo, Egypt; 2 Department of Anesthesia and Pain Management, Al-Fayoum University, Faiyum, Egypt; 3 Department of Anesthesia, Faculty of Medicine, King Saud University, Riyadh, Saudi Arabia; 4 Department of Surgery, Faculty of Medicine, Jazan University, Jazan, Saudi Arabia; 5 Department of Anesthesia, Intensive Care and Pain Management, Faculty of Medicine, Al-Azhar University, Cairo, Egypt; 6 Department of Anesthesia and Intensive, Faculty of Medicine, Luxor University, Luxor, Egypt

**Keywords:** Quadratus Lumborum Block, Intraperitoneal Bupivacaine Infiltration, Postoperative Analgesia, Laparoscopic Cholecystectomy

## Abstract

**Background:**

Inadequately managed acute pain following abdominal surgery can lead to patient discomfort, anxiety, respiratory issues, delirium, myocardial ischemia, prolonged hospital stays, and persistent pain.

**Objectives:**

This research compares the quadratus lumborum (QL) block to intraperitoneal and periportal bupivacaine infiltration for postoperative analgesia after laparoscopic cholecystectomy.

**Methods:**

This randomized double-blind study included seventy patients aged 21 to 60 years, randomly selected from Ain Shams University Hospital between March 2022 and March 2023, scheduled for elective laparoscopic cholecystectomy. The patients were divided into two groups of 35 based on postoperative pain management: Group A received intraperitoneal and periportal infiltration, while group B underwent the QL block, with details on placement and duration.

**Results:**

The results indicated a substantial decrease in Visual Analogue Scale (VAS) scores at 6 hours postoperatively, with group B exhibiting a median pain level of 3 [IQR 3 - 5] compared to group A's median of 5 [IQR 5 - 6], yielding a P-value of less than 0.001. The data indicate that group B had significantly enhanced postoperative analgesia within the initial 6 hours, both in a static condition and during movement. Moreover, patients in group B required fewer postoperative analgesics during the first 24 hours following surgery compared to group A.

**Conclusions:**

Following laparoscopic cholecystectomy, the QL block proved more effective than intraperitoneal and periportal bupivacaine infiltration in reducing postoperative pain scores for 6 hours and in reducing total opioid and analgesic use for 24 hours postoperatively.

## 1. Background

Uncontrolled postoperative pain can impede recovery, extend hospitalization, and elevate opioid consumption (1). A critical aspect of postoperative care is the implementation of effective pain management, especially following operations like laparoscopic cholecystectomy, which may induce both visceral and somatic pain (2). Postoperative pain after laparoscopic cholecystectomy is classified by anatomical region, specifically abdominal and shoulder discomfort according to location (3). Patients experience intense visceral discomfort caused by tissue damage and the stretching of nerve endings in the peritoneal cavity. Conversely, parietal discomfort results from incisional damage at the port sites, while diaphragmatic stretching characterizes shoulder discomfort (4).

One postoperative pain management technique is the quadratus lumborum (QL) block, in which a local anesthetic spreads to the paravertebral region to target the thoracolumbar fascia, providing analgesia that addresses both somatic and visceral pain pathways. Compared to other regional blocks, the QL block offers a wider range of pain relief due to its unique ability to anesthetize the lower thoracic and upper lumbar nerve roots. The QL block is effective; however, it requires technical expertise and ultrasound guidance for accurate anesthesia administration, which is not consistently feasible in all clinical environments. Although this block provides prolonged analgesia, its response is inconsistent, and challenges with deep needle insertion are common (5, 6).

Intraperitoneal local anesthetic is an effective therapeutic method, as its beneficial effects are closely associated with the pain characteristics following laparoscopic cholecystectomy, primarily induced by pneumoperitoneum. However, this technique is less effective in mitigating incision-related somatic pain (4, 7, 8). The effectiveness varies with the distribution and volume used. Despite these disadvantages, intraperitoneal infiltration is a simple and surgeon-friendly procedure that has yielded significant pain relief during the period immediately following surgery (9).

## 2. Objectives

The objective of this study was to assess the QL block against intraperitoneal and periportal local anesthetic infiltration for postoperative analgesia during laparoscopic cholecystectomy to determine which method more effectively reduced postoperative pain, narcotic consumption, and improved patient satisfaction.

## 3. Methods

This prospective, double-blind, randomized trial included patients scheduled for elective laparoscopic cholecystectomy under general anesthesia at Ain Shams University Hospital between March 8 and 9, 2023. The study was registered at ClinicalTrials.gov under the registration number NCT06721039 and was approved by the Ain Shams University, FMASU Faculty of Medicine's Research Ethics Committee (MS 168/2022). All procedures used in the study complied with the Declaration of Helsinki's guidelines. Prior to random assignment, each participant was required to complete an informed consent form.

Inclusion criteria comprised patients of both sexes, aged 21 to 60 years, undergoing elective laparoscopic cholecystectomy under general anesthesia, and categorized as having ASA physical status I or II. Exclusion criteria included patients who declined participation in the research, individuals with infections at the injection site, those with psychological issues resulting in non-cooperation, daily opioid users, symptoms of coagulopathy, and individuals exhibiting adverse reactions to the provided medications.

### 3.1. Randomization, Allocation and Masking

This randomized double-blind study, employing a stratified recruitment process to ensure balanced representation across subgroups, used computer-generated randomization and sealed envelopes to assign patients to two equal categories. Patients were blinded to the group assignment, as the four trocar sites and potential sites of needle insertion for the QL block were covered in all patients by a sterile dressing. Independent anesthesiologists performed the ultrasound-guided QL block and had no other role in the study. Surgeons who performed the intraperitoneal block had no role in outcome evaluation. Postoperative data and outcomes assessment were conducted by researchers who did not participate in the intraoperative anesthetic management and were unaware of the patients' group assignments.

Group A (n = 35) underwent general anesthesia, followed by intraperitoneal and port site bupivacaine infiltration. Group B (n = 35) underwent general anesthesia, followed by a lateral QL block.

### 3.2. Study Procedures

Patients were continuously monitored using non-invasive methods including arterial pressure, pulse oximetry, electrocardiography (ECG), temperature probe, and capnography. General anesthesia was induced through the establishment of two intravenous access sites, with the administration of 1.5 μg/kg fentanyl, 2 mg/kg propofol, and 0.5 mg/kg atracurium prior to endotracheal intubation. Mechanical ventilation was provided, targeting a tidal volume of 6 - 8 mL/kg based on the patient's ideal body weight, alongside a controlled respiratory rate to maintain an end-tidal carbon dioxide concentration within the range of 30 - 35 mmHg and End-expiratory pressure was consistently 5 cmH_2_O across all cases. Sevoflurane in a mixture of air and oxygen, along with 0.1 mg/kg atracurium, were administered to sustain anesthesia and preserve neuromuscular blockade. If the mean arterial pressure (MAP) fell below 65 mmHg, a 6 mg intravenous bolus of ephedrine was delivered. Atropine 0.6 mg IV was used to address a heart rate (HR) below 50 beats per minute.

After the surgery, 4 mg of dexamethasone was given intravenously, followed by 4 mg of ondansetron. The patient was then given the following analgesic technique:

- Group A: At the end of the operation, before the probes were removed, 20 mL of 0.25% bupivacaine was injected intraperitoneally (above the gallbladder, adjacent to and above the hepato-duodenal ligament, and into the hepato-diaphragmatic region). An additional 20 mL of 0.25% bupivacaine was administered in a divided manner to infiltrate the four trocar sites (7 mL for each of the two 10-mm trocar sites, and 3 mL for each of the two 5-mm trocar sites) (10).

- Group B: A radiopaque needle was inserted between the iliac wing and the 12th rib at the midaxillary line during a bilateral QL block, which was conducted with the patient in a lateral decubitus position. Using an ultrasonic probe, three layers of abdominal muscles were identified, and their posterior tracking allowed for the localization of the thoracolumbar fascia as well as the latissimus dorsi and other back muscles. The needle was inserted via an in-plane technique and progressed through the anterior abdominal musculature until it reached the anterolateral border of the QL. Precise insertion facilitates the distribution of local anesthetic between the QL and the middle layer of the thoracolumbar fascia, followed by the delivery of 20 cc of 0.25% bupivacaine for each side once the needle tip reaches the specified plane to improve hydro-dissection visualization and confirm the placement (11).

### 3.3. Post-Operative Settings

Upon completion of the procedure, the remaining neuromuscular blockade was reversed, and the patients were extubated. The Visual Analog Scale (VAS) score was recorded by a blinded researcher at 1, 2, 4, 6, 12, 18, and 24 hours postoperatively, while HR and blood pressure (BP) were measured at each specified interval. Patients exhibiting VAS ratings of 4 or higher at any time received morphine at a dosage of 0.5 - 2.5 mg every 10 - 30 minutes, with a maximum limit of 4 mg per hour administered intravenously.

#### 3.3.1. Primary Outcome

Postoperative pain evaluation was assessed using pain levels at rest and during mobilization. The VAS for pain, ranging from 0 (no pain) to 10 (highest pain), was assessed postoperatively every 2 hours for the initial 6 hours, followed by assessments every 6 hours for the subsequent 24 hours.

#### 3.3.2. The Secondary Outcomes

Postoperative hemodynamic parameters, including HR and MAP, were monitored. Additionally, the first time for rescue analgesia, morphine consumption in the first 24 hours, and the length of postoperative analgesia (the interval between recovery and the initial morphine dosage) were recorded.

### 3.4. Sample Size

We calculated the sample size using G*Power software, setting the power to 80% and the alpha error to 5%. Based on the results of previous studies, we assumed a medium effect size (0.3) for postoperative pain (12). The study's goal could be met with a sample size of at least 70 adult patients undergoing laparoscopic cholecystectomy, including 35 patients in each group.

### 3.5. Statistical Analysis

Data were analyzed using SPSS version 22.0. Parametric data were reported as mean ± standard deviation (SD), while nonparametric data were presented as median (interquartile range [IQR]). Qualitative data were expressed in terms of frequency and percentage. Continuous variables, including age, were analyzed using the *t*-test. The chi-square test was employed for categorical variables such as gender. A two-sided significance level of 0.05 and a 95% confidence interval were used for all effect tests. P-values were classified as significant when less than 0.05, very significant when less than 0.001, and non-significant when greater than 0.05.

## 4. Results

**Figure 1.**
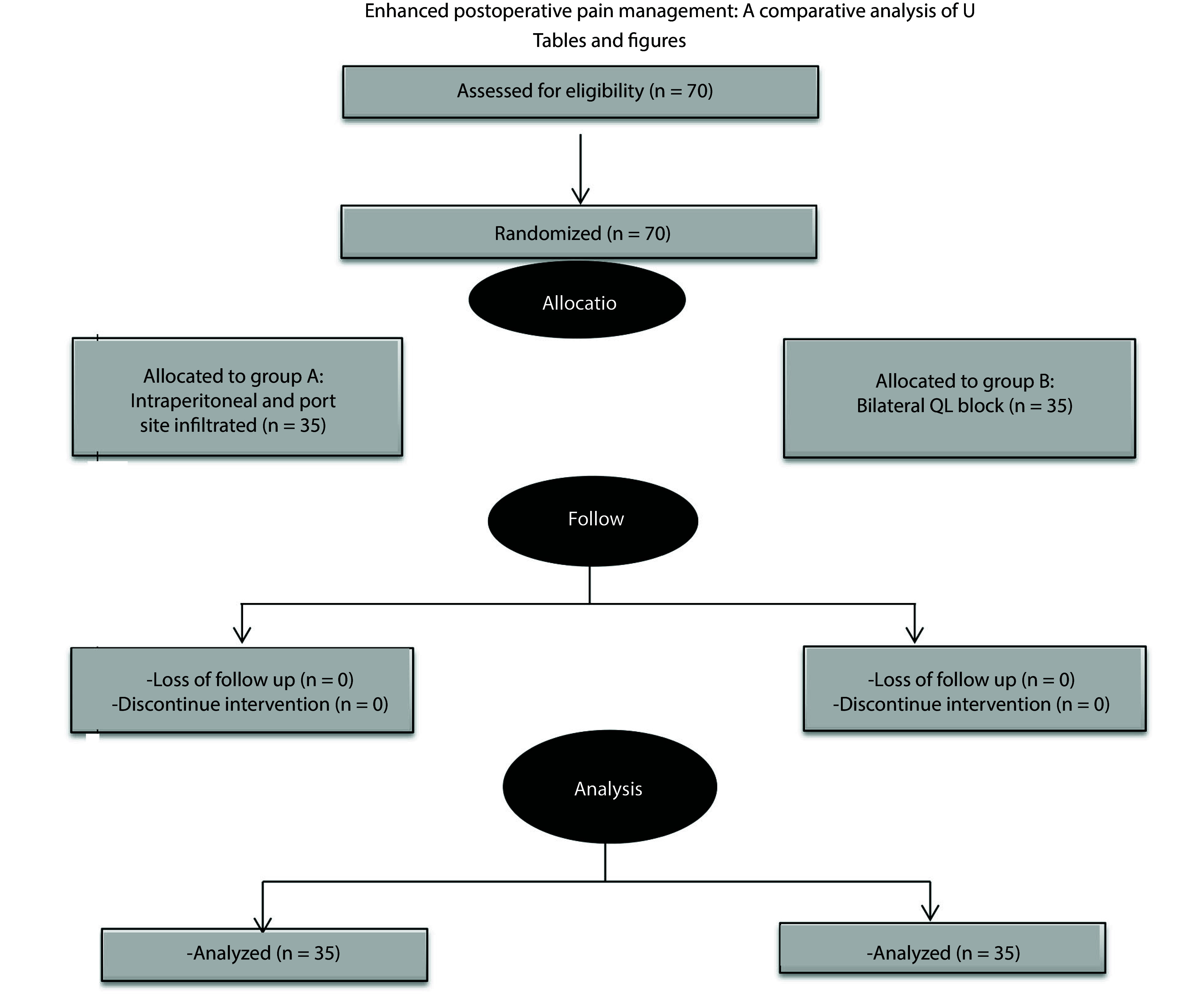
Consolidated standards of reporting trials (CONSORT) 2010 flow diagram illustrating the sequential phases of the trial

A total of 70 patients participated in the study conducted at Ain Shams University Hospital. The clinical study was conducted in accordance with the CONSORT flow diagram depicted in Figure 1. Recruitment concluded upon reaching the required sample size as determined by the sample size calculation. The demographic characteristics of patients, including age, BMI, sex, and ASA physical status, were statistically equivalent among the examined groups (Table 1). 

**Table 1.** Comparison Between Groups as Regards Demographic Data ^a^

Demographic Data	Group A (n = 35)	Group B (n = 35)	*t*; χ^2 ^ ^ b^	P-Value
**Age (y)**	43.26 ± 6.2	42.17 ± 6.7	*t *= 0.7	0.4856
**BMI (kg/m** ^**2**^ **)**	31.95 ± 2.9	31.59 ± 3.6	*t* = 0.5	0.64
**ASA**			χ^2^ = 0.23	0.63
I	19 (54.3)	17 (48.6)		
II	16 (45.7)	18 (51.4)		
**Gender**			χ^2^ = 0.0	1
Male	12 (34.3)	12 (34.3)		
Female	23 (65.7)	23 (65.7)		

^a^ Values are expressed as mean ± SD or No. (%).

^b^* t*, Student *t*-test; χ^2^, chi-square test.

Regarding MAP, although both groups exhibited approximately equivalent MAP values during the initial test, group A demonstrated significantly elevated MAP values from 2 to 6 hours compared to group B, with statistical significance. However, after 12 hours, the disparities in MAP became negligible as the values for both groups converged (Table 2). 

**Table 2.** Comparison Between Groups as Regards Mean Arterial Blood Pressure ^a^

MAP	Group A (n = 35)	Group B (n = 35)	*t * ^b^	P-Value
**MAB 1**	70.46 ± 7.5	70.63 ± 8.5	0.1	0.93
**MAB 2**	73.54 ± 6.8	70.54 ± 5.5	2.0	0.047
**MAB 4**	78.94 ± 6.4	72.63 ± 5.5	4.5	< 0.001
**MAB 6**	83.06 ± 4.3	76.17 ± 5.7	5.7	< 0.001
**MAB 12**	85.63 ± 5.6	82.91 ± 9.1	1.5	0.14
**MAB 18**	85.97 ± 3.4	84.60 ± 8.3	0.9	0.37
**MAB 24**	79.20 ± 4.8	78.11 ± 5.8	0.9	0.40

^a^ Values are expressed as mean ± SD.

^b^* t*, Student *t*-test.

Regarding HR, significant differences were observed between group A and group B from the 2-hour to 6-hour intervals, which were statistically significant; however, from the 12-hour mark onward, these differences nearly disappeared, with both groups exhibiting comparable HRs at the 24-hour mark (Table 3). 

**Table 3.** Comparison Between Groups as Regards Heart Rate ^a^

HR	Group A (n = 35)	Group B (n = 35)	*t * ^b^	P-Value
**HR 1**	78.29 ± 7.1	77.83 ± 7.3	0.3	0.79
**HR 2**	81.86 ± 5.4	75.66 ± 6.1	4.5	< 0.001
**HR 4**	86.43 ± 5.3	77.14 ± 6.1	6.8	< 0.001
**HR 6**	86.09 ± 5.6	79.20 ± 6.8	4.6	< 0.001
**HR 12**	87.40 ± 6.4	90.43 ± 10.3	1.5	0.14
**HR 18**	86.74 ± 4.2	89.26 ± 9.2	1.5	0.14
**HR 24**	82.91 ± 4.6	82.31 ± 6.3	0.5	0.65

^a^ Values are expressed as mean ± SD.

^b^* t*, Student *t*-test.

The pain levels of group A and group B were compared using the VAS at different time intervals. The baseline VAS 1 exhibited no significant differences across the groups. At VAS 2, group A exhibited a median pain score of 3 [IQR 2 - 4], while group B recorded a median score of 2 [IQR 2 - 3]. Group B experienced much less discomfort, with a median difference of 1, 95% confidence interval, 0 to 1; P = 0.005. The pattern persisted at VAS 4, with group A exhibiting a median score of 4 [IQR 4 - 5.75] and group B a median score of 3 [IQR 2.25 - 3], median difference of 2, 95% confidence interval, 1 to 2; with a significant P < 0.001, suggesting a consistent reduction in pain among group B. The disparity continued at VAS 6, with group A exhibiting a median pain level of 5 [IQR 5 - 6] and group B 3 [IQR 3 - 5], median difference of 2, 95% confidence interval, 1 to 2; yielding a P-value of less than 0.001. The data indicate that group B had significantly enhanced postoperative analgesia within the initial 6 hours. Prolonged trends indicate that median pain scores will align across groups. Over time, at VAS 12, 18, and 24, the disparity in pain levels decreased, resulting in comparable pain levels between both groups, which were no longer statistically significant (Table 4). 

**Table 4.** Comparison Between Groups as Visual Analogue Scale Score

Variables	Group A (n = 35)		Group B (n = 35)		P-Value ^a^
	Range	Median (IQR)	Range	Median (IQR)	
**VAS 1**	1 - 7	3 (2.0 - 3)	1 - 7	2 (1 - 3)	0.243
**VAS 2**	1 - 7	3 (2.0 - 4)	1 - 5	2 (2.0 - 3)	0.005
**VAS 4**	2 - 7	4 (4.0 - 5.75)	2 - 4	3 (2.25 - 3)	< 0.001
**VAS 6**	4 - 7	5 (5 - 6)	2 - 7	3 (3 - 5)	< 0.001
**VAS 12**	3 - 8	6 (5 - 7)	3 - 8	5 (4 - 7)	0.064
**VAS 18**	4 - 8	6 (5 - 6)	3 - 8	5 (4.25 - 6)	0.051
**VAS 24**	3 - 6	4 (4 - 5)	3 - 6	4 (3.0 - 5)	0.437

Abbreviation: VAS, Visual Analogue Scale.

^a^ Mann-Whitney test.

The total morphine consumption by patients in both groups indicated that group A required more morphine (15.40 ± 4.4) than group B (9.11 ± 3.2); mean difference, 6.29; 95% confidence interval, 4.45 to 8.13, demonstrating a highly significant difference between the groups, P < 0.001. The interval for rescue analgesia between the two groups indicated that group A required the rescue drug significantly earlier than group B, demonstrating a highly significant difference between them (Table 5). 

**Table 5.** Comparison Between Groups as Time for First Rescue Analgesia and Opioid Consumption ^a^

Variables	Group A (n = 35)	Group B (n = 35)	*t * ^b^	P-Value
**First rescue analgesia (min)**	209.14 ± 95.8	473.14 ± 246.0	5.9	< 0.001
**Total morph (mg)**	15.40 ± 4.4	9.11 ± 3.2	6.8	< 0.001

^a^ Values are expressed as mean ± SD.

^b^* t*, student *t* test.

## 5. Discussion

Laparoscopic cholecystectomy is one of the most common elective laparoscopic operations. Many patients experience right shoulder pain and incisional pain at the port sites after surgery (13). To provide postoperative analgesia following laparoscopic cholecystectomy, the current study compared the QL block to intraperitoneal and periportal infiltration with local anesthetics. This study demonstrated that the QL block is more successful in providing prolonged and efficient analgesia, as indicated by reduced VAS scores, accompanied by a significantly diminished total morphine dosage necessary within the initial 24 hours. The initial analgesic dose in the QL group was administered later than in the other group.

Our results confirmed the conclusions of Celik et al., who assessed the analgesic effectiveness of the QL block relative to wound infiltration for pediatric unilateral inguinal hernia repair. The results demonstrated a statistically significant difference in FLACC scores between the groups at the 2nd and 4th hours, although the differences at other time periods were not significant. Furthermore, the results indicated that the overall amount of analgesic use was reduced in the QL group after 24 hours (12).

Our research corroborated the findings of Kukreja et al., who demonstrated that the QL block considerably diminished VAS scores in patients relative to the control group following arthroplasty surgery. At 12 hours, the mean pooled VAS for the QL group was 2.34 (0.46), whereas the control group demonstrated a higher value of 3.33 (0.56). At 24 hours, the QL block group exhibited a mean of 2.31 (0.37), whereas the control group demonstrated a mean of 4.06 (0.42) (14).

Conversely, Vamnes et al. stated that the anterior QL block did not affect postoperative opioid usage when compared to a placebo block following laparoscopic cholecystectomy. They elucidated that no changes in opioid intake or postoperative discomfort were identified since muscle relaxants were not utilized, and only 20 mL of local anesthetics were administered for the QL block (15).

The research by Pandove et al. (13) claimed that the infiltration of bupivacaine at port sites with intra-abdominal infiltration successfully delivers postoperative analgesia and diminishes the need for postoperative analgesics. Saafan et al. also established that intraperitoneal bupivacaine improves pain scores and postoperative analgesia (16).

In our investigation, patients in the QL group required markedly less morphine postoperatively than those in the intraperitoneal and periportal bupivacaine infiltration groups. Elsharkawy et al. (5) demonstrated that patients undergoing a QL block required significantly less morphine for pain control compared to the local field infiltration (LFI) group. The total morphine intake was 9.11 mg ± 3.2 mg for the QL group, but the LFI group consumed 15.40 mg ± 4.4 mg.

Blanco et al. similarly examined the impact of the QL block on postoperative opioid utilization. Patients in the QL group had a marked reduction in opioid needs, with morphine intake about 40% lower than that of the LFI group, demonstrating the usefulness of the QL block. The reduction in morphine utilization within the QL group may enhance the benefits of the QL block by alleviating opioid side effects, thereby improving patient outcomes (11).

The current study indicates that the initial request for rescue analgesia in the QL block group was prolonged compared to the LFI group. The results are consistent with Dam et al., which demonstrated that the average time to the first request for rescue analgesia in the QL group was significantly extended. The QL block may offer extended analgesia by addressing both somatic and visceral pain pathways at a more proximal site (17). Blanco et al. observed analogous findings, indicating that the duration prior to the necessity for rescue analgesia was markedly extended in the QL block cohort (11).

Our results indicate a significant reduction in pain scores and opioid consumption with the use of the QL block, which delays the need for analgesia, supporting the QL block as an integral component of enhanced recovery after surgery (ERAS). This is consistent with the findings of Whiteley and Liu et al., suggesting that expanding ERAS protocols to include regional anesthesia beyond wound infiltration and the transverse abdominis plane (TAP) block may enhance postoperative pain control, recovery, and outcomes (18, 19). However, this is associated with additional cost implications of specialized equipment (ultrasound machine), training, and the time required for ultrasound-guided blocks compared to simpler techniques like local infiltration.

Our study had certain limitations, such as being a single-center study, lack of a control group, and not comparing postoperative side effects like postoperative nausea and vomiting in both groups. Additionally, the analgesic procedure was initiated at the end of surgery, and further studies to evaluate the preemptive effect of different approaches for the QL block on various surgical procedures are recommended.

### 5.1. Conclusions

The QL block demonstrated markedly greater efficacy than intraperitoneal and local anesthetic infiltration methods for the management of postoperative pain following laparoscopic cholecystectomy. The QL block resulted in reduced pain scores, decreased total morphine consumption, and an extended duration before the first rescue analgesia was administered.

## Data Availability

The dataset presented in the study is available on request from the corresponding author during submission or after publication.

## Clinical Trial Registration Code:

NCT06721039.
